# Gram-negative blood-stream infections and emerging antimicrobial resistance in children with acute myeloid leukemia during induction chemotherapy at Children’s Cancer Hospital Egypt 57357

**DOI:** 10.3389/fonc.2025.1540831

**Published:** 2026-04-22

**Authors:** Grace Mbatia, Reham Khedr, Leslie Lehmann, Mervat El Anany, Khaled Alsheshtawi, Omayma Hassanain, Sonia Ahmed, Omneya Hassanain, Lobna Shalaby, Alaa Elhaddad

**Affiliations:** 1 Department of Pediatric Hematology/Oncology Aga Khan University Hospital, Nairobi, Kenya; 2 Pediatric Oncology Department at Children’s Cancer Hospital Egypt 57357, Cairo, Egypt; 3 Pediatric Oncology Department at National Cancer Institute, Cairo University, Cairo, Egypt; 4 Department of Pediatric Hematology/Oncology, Stem Cell Transplantation, Dana-Farber Cancer Institute, Boston Children’s Hospital, Boston, MA, United States; 5 Department of Microbiology, Faculty of Medicine, Cairo University/Children’s Cancer Hospital Egypt 57357, Cairo, Egypt; 6 Department of Epidemiology and Biostatistics, Children’s Cancer Hospital Egypt 57357, Cairo, Egypt

**Keywords:** acute myeloid leukemia, induction chemotherapy, gram-negative blood-stream infections, multi-drug resistance, LMIC

## Abstract

**Background:**

In children receiving therapy for acute myeloid leukemia (AML), gram-negative bacteremia is associated with significant morbidity and mortality, especially in the presence of multi-drug resistant [MDR] organisms. objectives: The purpose of this study was to determine the prevalence and antibiotic susceptibility patterns of gram negativebloodstream infections [GNBSI]] during the period of induction chemotherapy [IC] and the associated morbidity and mortality in a large pediatric oncology hospital in a low- middle-income country (LMIC). patients and methods: This is a retrospective analysis of consecutive newly diagnosed patients with acute myeloid leukemia (AML) admitted for induction chemotherapy (IC) from January 2015 to December 2017 at the Children’s Cancer Hospital Egypt 57357.

**Results:**

We identified 90[27.4%] children with at least one GNBSI during induction, 72/90[80%] of whom had at least one MDR GNBSI. 124 GNBSI episodes were identified among the ninety children: *E.coli* 54.8%, *K.pneumoniae* 16.9%, *A.baumannii* 6.5%, *E.cloacae* 4.8%, and *P.aeruginosa* 4.0%. Thirty-day cumulative infection-related mortality was 27.8% [18.9-37.3%]. Associated morbidity included typhlitis [29/90 (32.2%)], sepsis-related central venous catheter [CVC] removal [12/90 (13.3%)], and admission to intensive care unit [38/90(42%)].

**Conclusion:**

Our study highlights the prevalence of gram-negative bacteria among febrile neutropenic children with AML and the high incidence of antibiotic resistance in our hospital. There is a need for keen surveillance globally of the epidemiology of multi-drug resistant gram-negative organisms and thoughtful studies into antibiotic stewardship to reduce the growing threat of MDR GN infections and the associated morbidity and mortality.

## Highlights

Antimicrobial resistant Bacteremia in Children with Acute Myeloid Leukemia during Induction Chemotherapy in LMIC.

## Introduction

Gram-negative bloodstream infections (GNBSI) are associated with high morbidity and mortality, especially in the immunocompromised host (1–3). During intensive induction chemotherapy (IC), children with AML are at increased risk of gram-negative bacteremia due to prolonged and often profound neutropenia, loss of mucosal integrity resulting in greater risk of translocation of gut bacteria into the bloodstream, and presence of indwelling intravenous catheters (1, 4–7). In recent years, there has additionally been growing global concern about the emergence of antibiotic resistance, resulting in difficult-to-treat infections with high case fatalities (8–11). The primary aim of this study was to describe the prevalence of infections and the pattern of antibiotic-resistant gram-negative organisms among children receiving induction chemotherapy for acute myeloid leukemia in a large free-standing pediatric oncology hospital in a low-middle income country (LMIC). The secondary aims were to determine the impact of antibiotic resistance in gram-negative bloodstream infections on patient outcomes with regard to morbidity and mortality and to describe the risk factors for mortality in the presence of antibiotic-resistant gram-negative infections. The primary outcome in this study was the 30-day mortality following microbiologically documented gram-negative bloodstream infection.

## Patients and methods

The institutional scientific medical advisory committee approved this study. Study Design:

This is a retrospective analysis of consecutive newly diagnosed patients with acute myeloid leukemia (AML) admitted for induction chemotherapy (IC) from January 2015 to December 2017 at the Children’s Cancer Hospital Egypt 57357, a tertiary pediatric oncology center in a low, middle-income country.

Excluded from the analysis were patients with Down syndrome (DS) and acute promyelocytic leukemia (APL). None of the patients during the study period developed AML as a secondary malignancy.

### Data collections

Patient bio-demographic, clinical, microbiological, laboratory, and radiological data is routinely collected by institutional clinical research associates (CRA) using standard data collection tools. Data were extracted from the hospital research department database. Institutional review board approval was obtained for this study.

### Treatment protocol

Patients were treated according to the institutional guidelines adopted from Arm A of the Children’s Oncology Group AAML 1031 study. Children received two courses of intensive induction chemotherapy. All patients received a first induction course of cytarabine + daunorubicin + etoposide (ADE). Patients with low-risk features received a second course of ADE, while those with high-risk features received a High dose of cytarabine and mitoxantrone (MA). Depending on physician preference, Chemotherapy was delivered through peripheral or central venous access.

### Antimicrobial therapy

Prophylactic levofloxacin for preventing bacterial infections was initiated for all patients on admission as part of standard policy. Dosing of Levofloxacin was as follows: <5 years: 10 mg/kg/dose every 12 hours. ≥5 years: 10 mg/kg/dose every 24 hours; maximum dose: 750 mg/dose.

Institutional guidelines recommended initiation of empirical Amikacin and Piperacillin/Tazobactum OR Carbapenem (in case of Extended Spectrum Beta-Lactamase [ESBL] organism infection within the preceding 6 weeks). A guideline change in November 2016 was instituted recommending the initiation of Amikacin and Carbapenem for all AML patients with FN.

### Work up

#### Microbiology

Blood samples for culture and sensitivity were drawn peripherally and from all lumens in patients with central venous catheters, and empiric antibiotics were administered for all children with fever and neutropenia [FN].

Automated blood culture (BC) systems used were; BD BACTEC^™^ 9240 Blood Culture System (Becton Dickinson), and the BacT/ALERT^®^ 3D System (bioMeriéux). Pediatric BC bottles (BACTECTM PedsPlusTM/F, BD Diagnostics, Shanon County, Clare, Ireland) and BacT/ALERT^®^PF Plus, INC. Durham), inoculated with 1–3 ml blood were incubated in the corresponding BC system. After reporting positive signal, positive blood culture broths were sub-cultured overnight according to standards, and colonies were identified by using MALDI-TOF (Vitex MS, bioMérieux). Antimicrobial susceptibility testing was done using the Vitek 2 system (bioMérieux). Also, manual susceptibility testing was done in parallel according to standards, and the results were interpreted according to CLSI guidelines. (12)

### Imaging

Abdominal Computed Topography (CT) scans were done for patients with suspected neutropenic enterocolitis (typhlitis) and only those with radiological evidence of disease were included in the analysis.

### Definitions

Fever and neutropenia were defined as per the Infectious diseases society of America (IDSA) guidelines:

Fever: a single oral temperature ≥38.3°C (101°F) or a temperature ≥38.0°C (100.4°F) lasting more than 1 hour, and Neutropenia: absolute neutrophil count (ANC) <500 cells/mm^3^ or an ANC that is expected to decrease to <500/mm3 during the next 48hrs (13).

Critically ill patients requiring supportive care with inotropes or mechanical ventilation those requiring supportive care with inotropes or mechanical ventilation and were transferred to the Intensive Care Unit (ICU). Blood samples were also drawn in patients with vital sign instability and hypothermic patients with temperatures below 35°C.

## Results

**Figure 1:**
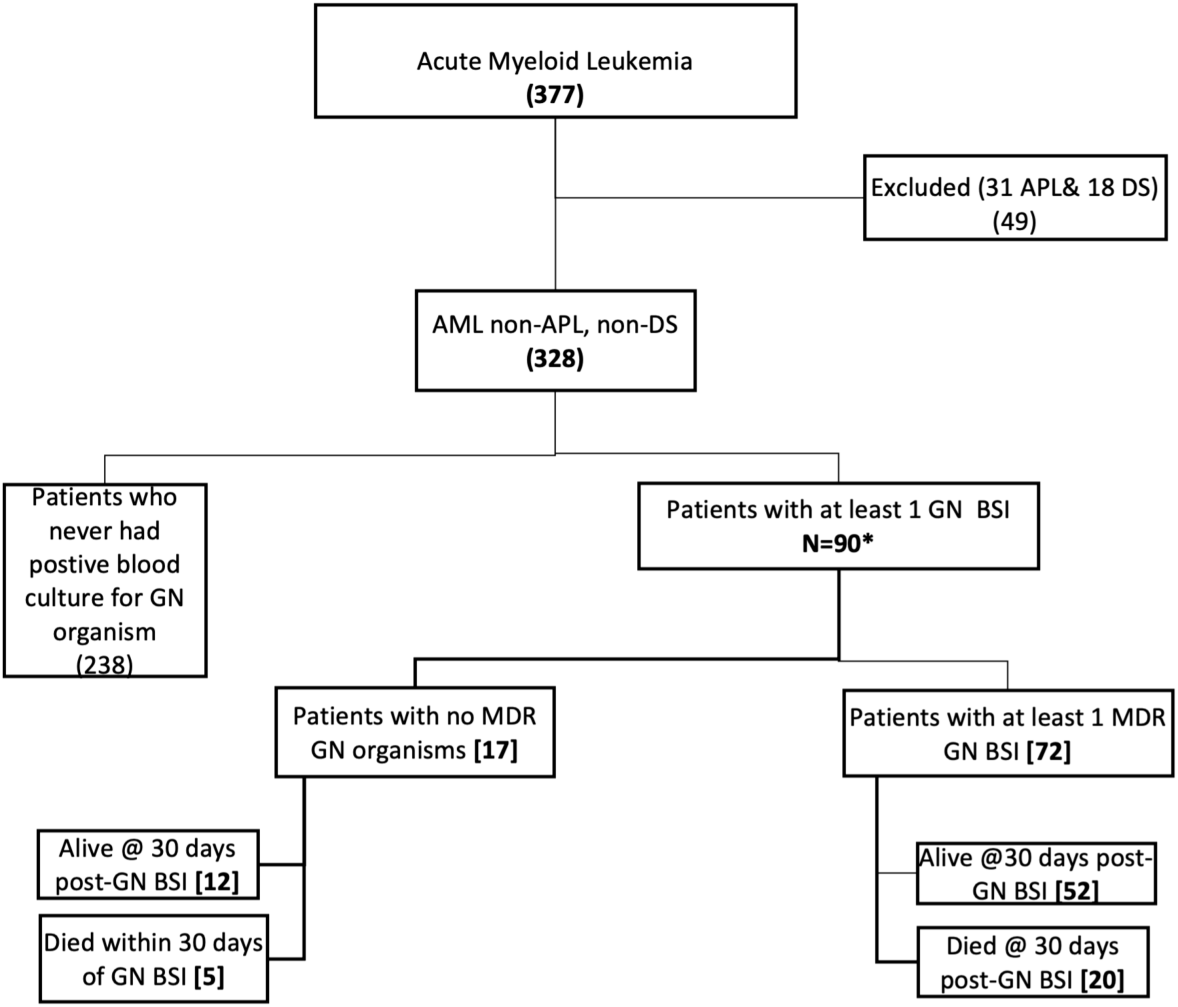
Study flow diagram. AML, Acute myeloid leukemia; APL, Acute promyelocytic Leukemia; DS, Down Syndrome; GN, gram-negative; MDR, Multidrug-resistant; GN BSI, Gram-negative Blood stream infection.

Three hundred and seventy-seven children were admitted for induction chemotherapy during the study period. After the exclusion of DS and APL AML patients, Blood culture results were reviewed for 328 patients. Ninety (27.4%) had at least one documented gram-negative BSI during the period of induction chemotherapy and were included in the analysis. Eighty percent (72/90) of these patients had at least one episode of a multi-drug resistant (MDR) gram-negative bacteremia (**Figure 1**).

The population’s median age was 8.1 years (0.4-17–8 years), and 68/90 (55%) were male. All patients received the first induction course of chemotherapy (ADE1) with no dose modifications. 20% of patients died during the 1^st^ course of therapy. Thus seventy-two (80%) received the second induction course: 38(42.2%) received ADE2 with no dose modifications, and 34 (37.7%) received intensified MA with three patients receiving modified chemotherapy (Idarubicin in place of Mitoxantrone) at the discretion of the attending physician.

A total of 124 GN BSI were observed among the ninety patients. Gram-negative bacteremia occurred in the setting of febrile neutropenia (FN) in 98% of cases (exceptions: two cases with fever and ANC> 500/µl at the time of blood culture and one in an afebrile patient where blood culture was obtained in the setting of severe abdominal pain). The median ANC on the first day of FN was 11/µl [range 2/µl -65.7/µl IQR 63/µl], and the mean temperature was 38.7°C with a fever range of 38.0°C to 40.0°C.

**Figure 2:**
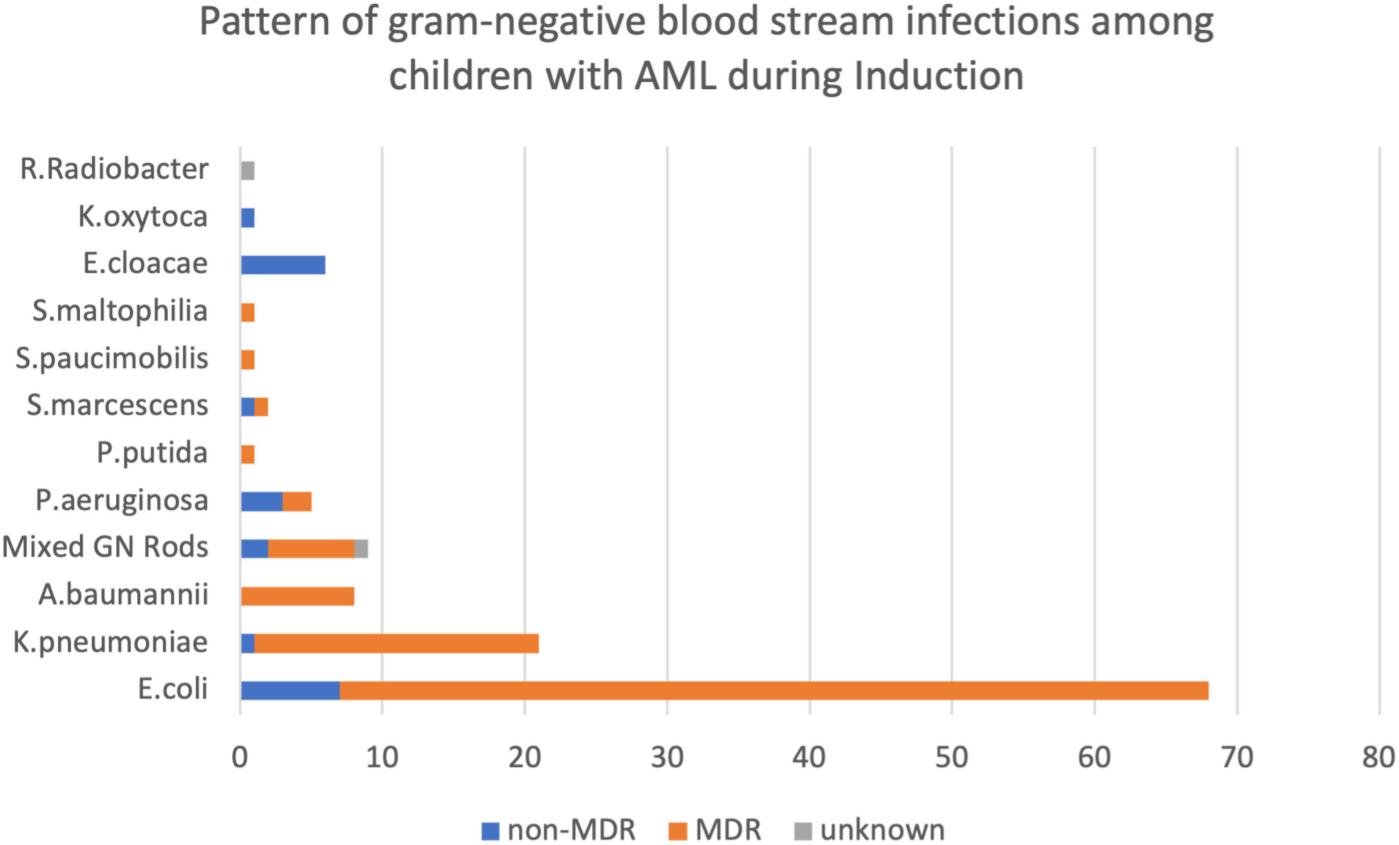
Pattern of gram-negative bloodstream infections among children with AML during induction. *E.coli, Escherichia coli; K. pneumoniae, Klebsiella pneumoniae; A. baumannii, Acinetobacter baumannii; E. cloacae, Enterobacter cloacae; P. aeruginosa, Pseudomonas aeruginosa; P. Putida, Pseudomonas Putida; S. Mascarenes, Serratia Mascarenes; S. Maltophilia, Stenotrophomonas Maltophilia; and K. Oxytoca, Klebsiella Oxytoca*.

Of the 124 GN BSI, 101 (81.45%) were with an MDR organism, 21 (17.7%) were non-MDR, and susceptibility testing was unavailable for two isolates. Among the isolated organisms, *Escherichia coli* (54.8%), Klebsiella pneumoniae (16.9%), Acinetobacter baumannii (6.5%), Enterobacter cloacae (4.8%), and Pseudomonas aeruginosa (4.0%) comprised the majority of the organisms. Other organisms included; mixed gram negative, *Pseudomonas Putida, Serratia Mascarenes, Stenotrophomonas Maltophilia, and Klebsiella Oxytoca*, (**Table 1** shows the antibiotic susceptibility of bacterial isolates, and **Figure 2** illustrates the distribution pattern of isolated organisms.)

**Table 1:** Antibiotic susceptibility patterns.

Antibiotic		*E. coli^1^*[68]	*Kleb p.^2^*[21]	Acineto b.*^3^*[8]	*MGN rods^4^*[9]	*Pseudo.aero.r^5^*[5]	*P.putida^6^*[1]	*Serratia m.^7^*[2]	*Sphingo p.^8^*[1]	*Steno m.^9^*[1]	*Entero c.^10^*[6]	*Klebo.^11^*[1]	Total [123]
Tigecycline	S	64	16	6	6		1	2	nil	1	6	1	103
	R	1		1		4	nil	nil	1	nil	nil	nil	7
	N	3	5	1	3	1	—	—	—	—	—	—	13
Colistin	S	67	21	8	8	5	1		1	1	6	1	119
	R		nil	nil		nil	nil	1	nil	nil	nil	nil	1
	N	1	—	—	1	—	—	1	—	—	—	—	3
Meropenem	S	22	4	nil	3	2	nil	2	nil	nil	6	1	40
	R	46	17	8	5	2	1	Nil	1	1	nil	nil	81
	N	—	—	—	1	1	—	—	—	—	—	—	2
Imipenem	S	24	4	nil	3	2	nil	1	nil	nil	6	1	41
	R	41	17	8	4	3	1	1	1	1	nil	nil	77
	N	3	—	—	2	—	—	—	—	—	—	—	5
Amikacin	S	53	12	1	5	3	1	1	1	nil	6	1	84
	R	15	9	7	3	2	nil	1	nil	1	nil	nil	38
	N	—	—	—	1	—	—	—	—	—	—	—	1
Piperacillin/Tazobactam	S	3	1		1	2	nil	1	nil		2	1	11
	R	54	18	7	7	3	1	1	1		4	nil	96
	N	11	2	1	1	—	—	—	—	1	—	—	16
Ciprofloxacin	S	1	1		2	2	nil	1	1	nil	5	nil	13
	R	64	20		6	2	1	1	nil	1	1	1	105
	N	3	—		1	1	—	—	—	—	—	—	5
Cefepime	S	3	1	nil	3	2	nil	1	nil	nil	2	1	13
	R	64	20	8	5	3	1		1	1	4	nil	107
	N	1	—	—	1	—	—	1	—	—	—	—	3
Ceftriaxone	S	2		nil			nil	1	nil	nil	1	1	5
	R	61	19	8	7	4	1	1	1	1	5	nil	108
	N	5	2	—	2	1	—	—	—	—	—	—	10
Ceftazidime	S	2	nil	nil	1	2	nil	1	nil	nil	2	nil	8
	R	65	21	8	7	3	1	1	1	1	4	1	113
	N	1	—	—	1	—	—	—	—	—	—	—	2
Cefotaxime	S	1					nil	1	nil	nil	1	1	4
	R	63	20	7	8	4	1	1	1	1	5	nil	111
	N	4	1	1	1	1	—	—	—	—	—	—	8
Cefperazone/Sulbactum	S	3			1	2		1	nil		2	1	10
	R	38	18	7	7	3		1	1		4	nil	79
	N	27	3	1	1	—	1	—	—	1	—	—	34
Trimethoprim/Sulfamethoxazole	S	3	3		1	nil	nil	1	nil	nil	nil	1	9
	R	62	15	7	7	5	1		1	1	6	nil	105
	N	3	3	1	1	—	—	1	—	—	—	—	9

S, sensitive; R, resistant; N, neutral; *E.coli, Escherichia coli; K. pneumoniae, Klebsiella pneumoniae; A. baumannii, Acinetobacter baumannii; E. cloacae, Enterobacter cloacae; P. aeruginosa, Pseudomonas aeruginosa; P. Putida, Pseudomonas Putida; S. Mascarenes, Serratia Mascarenes; S. Maltophilia, Stenotrophomonas Maltophilia; and K. Oxytoca, Klebsiella Oxytoca.*

### Mortality

**Figure 3:**
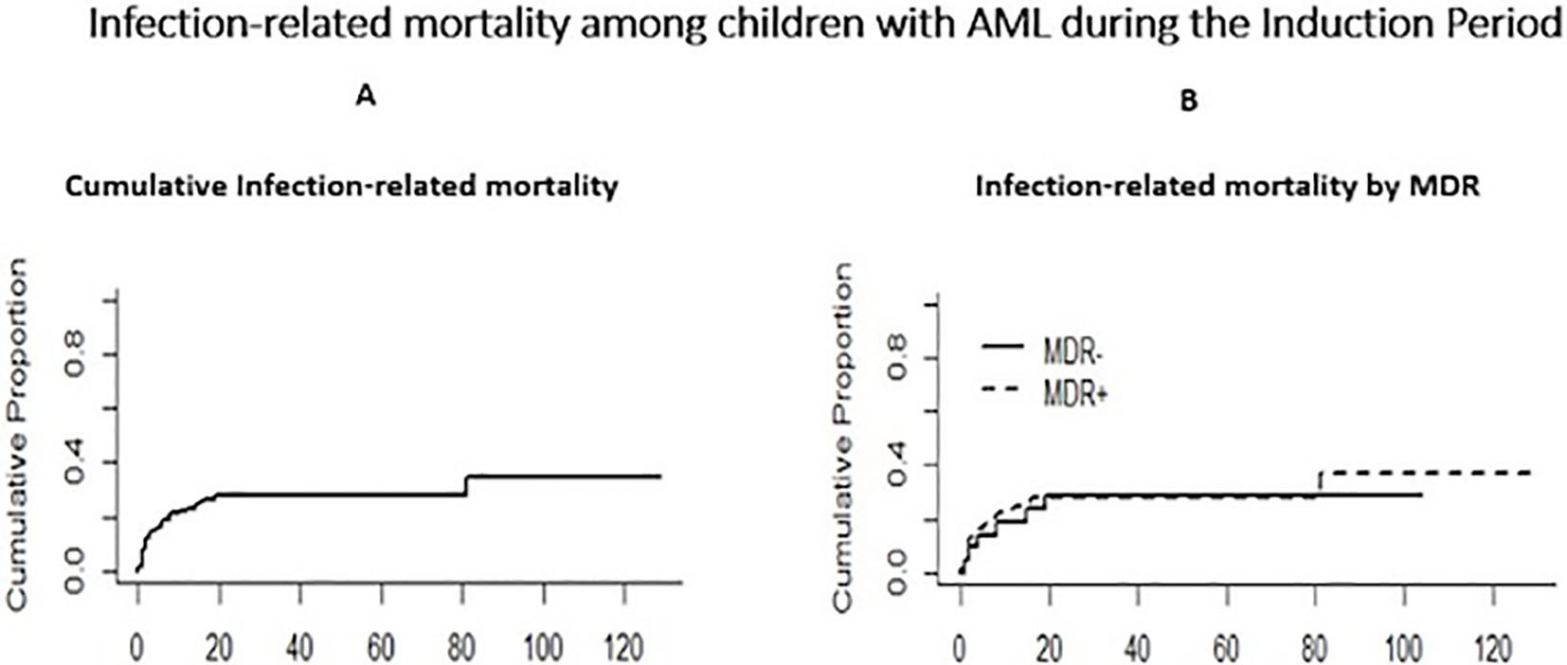
Infection-related mortality. AML, Acute myeloid leukemia; MDR, Multidrug resistant.

The overall survival (OS) of the AML patients during the study period was OS 49.44% and the EFS was 44.17%. Twenty-six (28.8%) of the 90 children with a documented GNBSI died during the induction courses, 18(20%) and 8(8.8%) during the first and second induction periods, respectively. Almost all mortalities occurred within thirty days of a documented GNBSI (25/26). We evaluated mortality at 48 hours, 7 days, and 30 days from the onset of the febrile neutropenic episode with positive blood culture for gram-negative organisms. The cumulative infection-related mortality at 48 hours, 7 days, and 30 days, overall, and by MDR status were not statistically significant and are shown in **Table 2** and **Figure 3** below. The cumulative incidence of infection-related mortality among children with MDR GN infection versus those with susceptible GN infection was not statistically significant, 27.9% (17.8%-38%) versus 28.6% (11.3%-48.7%), respectively.

**Table 2:** Cumulative incidence of infection-related mortality.

Time point (days)	Cumulative Incidence of Infection related Mortality	Cumulative Incidence of Infection related Mortality MDR (Y)	Cumulative Incidence of Infection related Mortality MDR (N)
2	12% (6%-20%)	13% (6.4%-22.5%)	10% (1.5%-26.6%)
7	18.9% (11.6%-27.6%)	20.6% (11.9%-30.9%)	14.3% (3.4%-32.6%)
30	27.8% (18.9%-37.3%)	27.9% (17.8%-38%)	28.6% (11.3%-48.7%)

MDR, multidrug resistant

Only 2/26 deaths occurred outside the ICU setting; one patient died in the ward due to sudden pulmonary hemorrhage, and one had suspected typhlitis and sepsis. Ninety-two percent of the deaths occurred in the ICU [24/26] and were associated with septic shock [16], severe respiratory distress [4], post-operative typhlitis with ruptured viscus [1], altered level of consciousness with positive image findings of intracranial hemorrhage [1], cardiac failure [1] and upper airway obstruction [1].

### Morbidity

In terms of morbidity among the 90 patients with GNBSI, we captured the need for central venous catheter (CVC) removal in those with CVC, ICU admission, and radiological evidence of typhlitis. Seventeen of the ninety (18%) children had CVC placement during the induction period. Thirteen of the seventeen (76.5%) had CVC removal during induction. The indication for CVC removal was associated with central-line infection in all thirteen patients. CVC re-insertion during IC was done for only one of the thirteen patients with initial episodes of documented *Klebsiella pneumoniae* and subsequent *Escherichia coli* bacteremia. Re-insertion of a CVC was done three weeks after the last documented infection. However, subsequent removal of the second CVC was done in this patient after a blood culture showed persistent *Escherichia coli* infection two weeks after catheter re-insertion.

Thirty-eight (42%) patients had at least one admission to the Intensive care unit (ICU): 35/90 had a single ICU admission and 3/90 had two ICU admissions. Among the children with two ICU admissions, one was admitted both times for upper airway obstruction in the setting of mucositis with severe respiratory distress and subsequent respiratory failure; the other two were admitted due to sepsis on both occasions. The reasons for admission among the thirty-eight children were: sepsis [24]; post-operative typhlitis with ruptured viscus [1]; severe respiratory distress [5]; altered level of consciousness with positive image findings of intracranial hemorrhage [1]; altered level of consciousness with hypertension [1]; congestive cardiac failure [3]; cardiac arrhythmia [2] and upper airway obstruction [1]. Half of the children [19/38] admitted to ICU received inotropic support, 22/38 required mechanical ventilatory support and 24/38 died as detailed above under the ‘mortality’ section. The median duration for ICU admission among those who died was 6.5 days (range = 1–88 days, mean=13.3 days) versus 3 days (median=3 days, range = 2–10 days, mean= 5 days) for those who survived.

Radiological evidence of typhlitis was seen in 29 (32.2%) patients, 23 in the first and 6 in the second Induction periods, respectively. Six patients required laparotomy. **Table 3** reports details of the surgical intervention.

**Table 3:** Typhlitis in children with gram-negative bloodstream infections during AML induction chemotherapy.

Patient	Laparotomy findings	Surgical intervention	Outcome
1	perforated gangrenous intestine; proximal jejunum and descending colon with necrotic transverse colon	complete excision/colostomy	Dead
2	patchy discoloration of small bowel	Use of warm packs-small bowel	Dead
3	intra-peritoneal purulent collection with perforation at terminal ileum, lacerated right colon	complete excision/primary anastomosis	Alive
4	fecal peritonitis with pyogenic membranes covering bowel and peritoneum	hemicolectomy	Alive
5	atonic segment of proximal jejunum with patchy discoloration	Use of warm packs resection/primary anastomosis and Infra-colic omentectomy	Dead
6	gangrenous jejunum with inflammation at caecum	resection/primary anastomosis	Alive

### Neutrophil recovery

**Figure 4:**
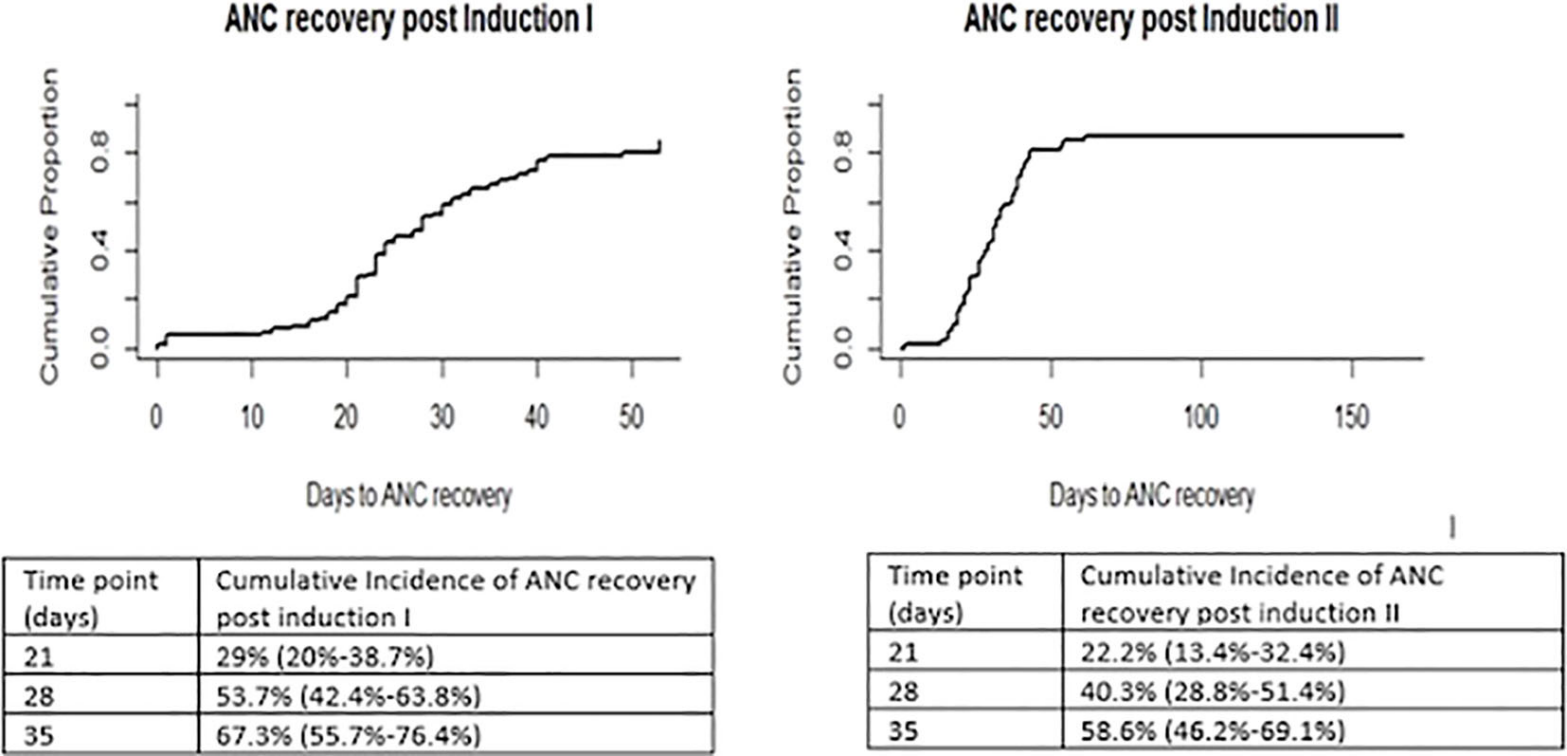
Time to neutrophil recovery. ANC, absolute neutrophil count.

The time to neutrophil recovery is illustrated in **Figure 4**. The cumulative incidence of neutrophil recovery at day 28 post-induction I was 53.7% (42.4%-63.8%) versus 40.3% (28.8%-51.4%).

## Discussion

Historically, treatment-related mortality (TRM) in pediatric AML was high, mostly due to infectious complications (14, 15). Long-term survival rates approach 70%, and TRM has declined significantly in developed countries (16–19). Improvements in both early outcomes and long-term survival have been attributed to optimal risk stratification, intensified chemotherapy and stem cell transplantation for high-risk patients, and continuous advances in supportive care (20, 21). TRM had decreased due to increased attention to infectious risks and the use of prophylactic antibiotics (20). Despite these advances, infectious complications and the emergence of multidrug-resistant organisms could undermine previous gains and remain particularly problematic in patients treated in LMIC (1, 22).

The lack of access to modern diagnostic tools and adequate supportive care resources further complicates the management of AML in LMICs, leading to delays in diagnosis and treatment that can result in disease progression and increased mortality (23, 24).

Socioeconomic disparities and barriers to healthcare access significantly contribute to treatment abandonment and refusal, resulting in preventable deaths (25).

Families often face financial constraints, limited availability of essential medications, and inadequate counseling regarding treatment options, which undermine their ability to pursue effective care (26–28).

Many children in this cohort had delayed diagnosis leading to lack of timely interventions, and inadequate supportive care that lead to worse outcomes compared to high-income countries (HIC).

Addressing these systemic inequities is crucial for improving the prognosis for children with AML, as evidenced by the disparity in five-year overall survival rates between LMICs and high-income nations, where rates can be as low as 5% compared to upwards of 60% in more developed healthcare systems (27, 28).

Also, prolonged and/or severe neutropenia increases susceptibility to infections, particularly in children undergoing intensive chemotherapy (29, 30). Our study showed that the median absolute neutrophil count (ANC) at the onset of febrile neutropenia was low, which likely contributed to the high incidence of GNBSI and subsequent mortality. In a study of 302 patients with congenital neutropenia treated with granulocyte colony-stimulating factor (G-CSF), the mean duration of neutropenia was observed to be 14 days, highlighting the prevalence of this condition among pediatric patients (31, 32).

Moreover, over 40% of infectious complications occurred during induction therapy, emphasizing the vulnerability of patients during this critical treatment phase (33).

Patterns of organisms responsible for infectious complications vary according to the time period of study, regional socio-economic factors, and advances in healthcare (22). In recent decades, gram-negative bacteria have been shown to be responsible for a significant proportion of BSI in FN children (11, 22) and are associated with worse clinical outcomes (2, 11). Rapidly growing antibiotic resistance further complicates the treatment of children experiencing FN during AML therapy (9, 34, 35).

This emerging problem has not been studied in detail in low middle income countries (LMIC) where access to antibiotics, antibiotic usage and susceptibility patterns could be expected to be different. In Egypt, antibiotics are readily available and can be obtained from community pharmacies without prescriptions, even though dispensing antibiotics without a prescription is against Egyptian law (36). Responsible antibiotic use is crucial for preserving their effectiveness and combating antimicrobial resistance.

Our cohort comes from a center that treats only pediatric oncology patients from throughout Egypt. We found a high rate of gram-negative bacteremia in our population, compared to that reported in the United States (37).Twenty-seven percent (90/328) of children had at least one GNBSI during IC and there were 124 unique episodes of GNBSI among the 90 children. Surprisingly and concerningly, 101 (81.45%) were MDR. A prospective multi-institutional study of 492 children with AML from the Children’s Oncology Group found lower rates of GNBSI (37). Similar to our study, this study excluded patients with acute promyelocytic leukemia, Down syndrome, or AML as a second malignancy and the induction regimen used was idarubicin, daunomycin, cytarabine, thioguanine, etoposide, and dexamethasone [IdaDCTER]). Eighteen percent (87/492) of children had at least one documented GNBSI and the cumulative incidence of infection-related mortality was 11% plus or minus 2% during chemotherapy administration excluding stem cell transplant. However, this study was carried out prospectively, in an earlier era with analysis through to June 2006 and analysis of MDR was not undertaken.

In another study from Europe, children treated according to the multi-institutional clinical trial AML-BFM 93 (38), 855 infectious complications were observed in 304 patients (fever without identifiable source (n=523; 61.2%), clinically (n=57; 6.7%) and microbiologically documented infections (n=275; 32.1%). Blood stream infections accounted for 228 of the 275 microbiologically documented infections. In this study the majority of infections were due to gram positive organisms 88.5% (202/228) versus 18.4% (42/228) gram-negative organisms. This study included patients with Down syndrome. Chemotherapeutic agents were similar to those in our study population (ADE and MA), This study was carried out in an earlier era and the analysis was not limited to the induction period. Despite the different inclusion/exclusion criteria, there is a clear difference from our results which showed predominantly gram-negative BSI.

In the later AML-BFM 2004 trial (39), 405 patients experienced infectious complications with 32.4% having a microbiologically documented infection. This study excluded patients with Down syndrome. Gram-positive organisms were isolated in 72.7% (240/330) versus GNBSI in 27.2% (90/330). In this study, there was a significantly reduced infection related mortality (1.5% versus 5.4% *P*=0.003) compared to the earlier AML-BFM 93 study possibly attributable to specific antimicrobial prophylaxis and improved supportive care.

In terms of studies from LMIC, a study carried out in India among children 1–18 years receiving IC for AML with a three-drug regimen (cytarabine + daunorubicin + etoposide) reported 96 episodes of FN among 54 children (40). Blood cultures were positive in 19% (18/96) and GN bacteria were isolated in 90% (16/18). The study spanned a 13-year period compared to only 3-year period of our study and had a smaller population. The authors divided the study period into two cohorts due to a shift in the resistance patterns of GN organisms (cohort 1: from 2002 to 2011, cohort 2: from 2012 to 2016). The time period for cohort 2 overlaps with our study. During cohort 2 study period, the authors reported a statistically significant surge in multidrug-resistant (MDR) infections, 87% of organisms showed *in-vitro* resistance to carbapenems compared to only 20% during cohort 1. We found 65.8% (81/123) *in vitro* resistance to meropenem in our study. Although our study included only gram-negative organisms, we see an alarmingly high rate of antibiotic resistance which may point to growing global trends that may be reflected in our region in coming years. However, it is noted that the number of documented blood cultures is much smaller (16 GNBSI versus 90 GNBSI). The mortality in the India study was 10% (5/54) at the end of two cycles of induction chemotherapy. Sepsis was reported as the primary cause of death in all five children and 80% (4/5) had a MDR gram negative organism.

The mortality among children with GNBSI in our study was 28.8% during IC. Infection-related mortality in our study is higher than that reported in other AML studies (37, 39–42). The reason for higher infection-mortality rates in our study could be we included only patients with a gram-negative bacteremia, known to be associated with high case fatalities and the use of highly myelosuppressive induction chemotherapy regimen. It is also plausible that our population might have a higher burden of infectious disease than patients in the MRC AML10, CCG2961 and AML-BFM 93 and 2004 BFM trials.

Despite the high prevalence of MDR in our study population, the impact on mortality was not statistically significant with similar 30-day cumulative incidence of infection related mortality 27.9% in the MDR group versus 28.6% in the non-MDR group *p* = 0.86. Use of broad-spectrum antibiotics and highly intensified supportive care measures in patients with MDR GNBSI are likely reasons for this observation. However, with the constant threat of newly emerging resistant gram-negative organisms, the armamentarium of antibiotics is declining and new strategies are needed to maintain survival among patients with MDR GNBSI (43). This will be particularly important in LMIC as improvements in the global delivery of pediatric oncology care result in more centers in more countries delivering therapy for AML.

Although no difference in mortality was demonstrated, there was significant morbidity noted in our population and intense resource utilization: 42% (38/90) had at least one ICU admission, 76.5% (13/17) of children with CVC and GNBSI required catheter removal, and catastrophic gastrointestinal morbidity was demonstrated in 32.2% (29/90) with radiological evidence of necrotizing enterocolitis.

Neutropenic enterocolitis (NEC or typhlitis) is a serious and potentially life-threatening complication seen in patients undergoing intensive treatment with chemotherapeutic agents which can cause severe mucositis, neutropenia and impaired immunologic function. (44) We noted a prevalence of 32.2% (29/90) of NEC in our population during the induction period. A study carried out in Turkey among children with hematological malignancies which defined NEC as the presence of the clinical triad of abdominal pain, fever and neutropenia or imaging signs (thickened bowel wall) plus two of the clinical features demonstrated a cumulative incidence of typhlitis of 7.4% in patients with ALL and 28.5% in patients with AML or bi-phenotypic leukemia (45) for the complete duration of therapy. It is probable that we might have observed higher rates of NEC if we included patients with clinical symptoms only and did not limit analysis to the induction period only. NEC-associated mortality is very high, ranging from 50% to 100%. Six of the 29 children with NEC required surgical intervention, and of these 50% (3/6) died. Overall, 27.5% (8/29) children with NEC died during the induction period.

Our study highlights the prevalence of gram-negative bacteria among febrile neutropenic children with AML and the high incidence of antibiotic resistance in our hospital. We also found that while we did not demonstrate that early mortality was increased, there was significant morbidity in terms of the need for ICU-level care, neutropenic enterocolitis, and CVC removal.

Over the last few years, we have established and executed our antimicrobial stewardship initiatives at the institutional levels to monitor and evaluate antimicrobial usage to ensure optimal and responsible practices. We have Implemented surveillance protocols for new admissions to identify potential colonization since early detection can help prevent the spread of resistant bacteria. Also, we used our local antibiograms to guide empiric (initial) and targeted (specific) antibiotic treatments and to tailor therapy based on local resistance patterns.

There is a need for keen surveillance globally of the epidemiology of multi-drug resistant gram-negative organisms and thoughtful studies into antibiotic stewardship to reduce the growing threat of MDR GN infections and the associated morbidity and mortality. However, our study does have certain limitations. It is a retrospective study, and we could not obtain certain microbiological data, such as the time to positivity of cultures and molecular data for detecting resistance genes.

## Data Availability

The raw data supporting the conclusions of this article will be made available by the authors, without undue reservation.
